# The expression of the chemokine receptor CCR5 in tick-borne encephalitis

**DOI:** 10.1186/s12974-016-0511-0

**Published:** 2016-02-22

**Authors:** Sambor Grygorczuk, Joanna Osada, Miłosz Parczewski, Anna Moniuszko, Renata Świerzbińska, Maciej Kondrusik, Piotr Czupryna, Justyna Dunaj, Milena Dąbrowska, Sławomir Pancewicz

**Affiliations:** Department of the Infectious Diseases and Neuroinfections, Medical University of Białystok, ul. Żurawia 14, 15-540 Białystok, Poland; Department of Hematologic Diagnostics, Medical University of Białystok, ul. Waszyngtona 15A, 15-269 Białystok, Poland; Department of Infectious Diseases and Hepatology, Pomeranian Medical University in Szczecin, ul Arkońska 4, 71-455 Szczecin, Poland

**Keywords:** Tick-borne encephalitis, Th lymphocytes, Chemokines, CCR5, *CCR5Δ32*

## Abstract

**Background:**

Chemokine receptor 5 (CCR5) is hypothesized to drive the lymphocyte migration to central nervous system in flavivirus encephalitis, and the non-functional *CCR5Δ32* genetic variant was identified as a risk factor of a West Nile virus infection and of tick-borne encephalitis (TBE). We have attempted to investigate how CCR5 expression corresponds to the clinical course and severity of TBE.

**Methods:**

We have repeatedly studied CCR5 expression in 76 patients during encephalitic and convalescent TBE phase, analyzing its association with clinical features, cerebrospinal fluid (csf) pleocytosis, and concentrations of CCR5 ligands (chemokines CCL3, CCL4, and CCL5) and *CCR5* genotype. Fifteen patients with neuroborreliosis, 7 with aseptic meningitis, 17 in whom meningitis/encephalitis had been excluded, and 18 healthy blood donors were studied as controls. Expression of CCR5 was measured cytometrically in blood and csf-activated Th lymphocytes (CD3+CD4+CD45RO+). Concentrations of chemokines in serum and csf were measured immunoenzymatically, and *CCR5Δ32* was detected with sequence-specific primers. Data were analyzed with non-parametric tests, and *p <* 0.05 was considered significant.

**Results:**

The blood expression of CCR5 did neither differ between the groups nor change in the course of TBE. The CCR5 expression in the inflammatory csf was several-fold increased in comparison with blood but lower in TBE than in neuroborreliosis. The csf concentration of CCL5 was increased in TBE, the highest in the most severe presentation (meningoencephalomyelitis) and correlated with pleocytosis. The *CCR5Δ32/wt* genotype present in 7 TBE patients was associated with a decreased CCR5 expression, but enrichment of csf Th population in CCR5-positive cells and the intrathecal inflammatory response were preserved, without a compensatory increase of CCL5 expression.

**Conclusions:**

We infer CCR5 and CCL5 participate in the response to TBE virus, as well as to other neurotropic pathogens. The intrathecal response to TBE is not hampered in the bearers of a single copy of *CCR5Δ32* allele, suggesting that the association of *CCR5Δ32* with TBE may be mediated in the periphery at the earlier stage of the infection. Otherwise, a variability of the CCR5 expression in the peripheral blood lymphocytes seems not to be associated with a variable susceptibility to TBE.

## Background

Tick-borne encephalitis (TBE) is caused by a tick-borne encephalitis virus (TBEV) of *Flavivirus* genus (family *Flaviviridae*), transmitted by *Ixodes* ticks. It is endemic in the temperate zone of Asia and Eastern and Central Europe, where several thousand cases are reported annually, including over 200 cases in Poland [1–3]. Infection with the European TBEV type is often asymptomatic or results in a mild, flu-like disease. The second phase of the infection, characterized by central nervous system (cns) involvement, occurs in a minority of cases and presents an uncomplicated meningitis, meningoencephalitis of highly variable severity or meningoencephalomyelitis [4–8]. This wide range of clinical presentations suggests that host factors contribute to the susceptibility to the disease and to its severity. The outcome of the infection may be decided at several points, including the local virus replication and spread from the tick-bite site, the peripheral response immune/inflammatory, the penetration of the blood-brain barrier, and finally, the type and extent of the response within cns [9]. Nervous tissue pathology in TBE is complex and involves both the direct cytopathic effect of TBEV on the infected neural cells and the secondary immune-mediated damage [9–13].

The cerebrospinal fluid (csf) pleocytosis is a hallmark of the intrathecal inflammatory response during cns infection. In TBE, the lymphoid pleocytosis is relatively low and dominated by Th CD4+ lymphocytes, mostly of Th1 subset, with addition of T CD8+ cells [14–16]. The role played by different cell populations, including T CD4+ and T CD8+ lymphocytes, is debatable [17]. Both animal experiments and autopsy studies in fatal human TBE cases suggest Th CD4+ lymphocytes are indispensable for the TBEV control and elimination while Tc CD8+ cells contribute to cns immunopathology [13, 18]. Leukocyte migration into cns is driven by cytokines of the chemokine family, and a pattern and timing of expression of particular chemokines and their receptors determines the composition of the infiltrate [19, 20]. Chemokine receptor 5 (CCR5) is a receptor for chemokine ligand 3 (CCL3, previously named MIP-1α), CCL4 (MIP-1β), and CCL5 (RANTES) expressed on memory type and activated Th1 CD4+ lymphocytes, T CD8+ lymphocytes, and monocytes, and involved in Th1-type inflammatory/immune response [21–25]. Its expression is not increased directly on lymphocyte activation but is up-regulated by pro-inflammatory and Th1-related cytokines (interleukin 2—IL-2, IL-12, tumor necrosis factor alpha—TNF-α, interferon gamma—IFN-γ) and may be decreased in anti-inflammatory/Th2 environment [21, 24, 25]. Several studies show increased CCR5 expression by lymphocytes in the inflammatory csf [26–30], but the data on its role in flavivirus encephalitis are mostly indirect.

The *CCR5Δ32* deletion in *CCR5* gene results in a synthesis of a truncated protein and the lack of functional CCR5 in homozygotes [24, 31]. In *CCR5Δ32* heterozygotes, both the constitutive expression of CCR5 and its induction by pro-inflammatory stimuli are significantly reduced [24, 25]. The lack of CCR5 confers no evident pathology, probably due to a redundancy within the chemokine ligand/receptor network, but it hampers protective responses against certain viral and parasitic pathogens in animal models [31–34]. In humans, impaired CCR5 function has been suggested as a risk factor of flavivirus infections. A case of a severe viscerotropic disease after vaccination with an attenuated yellow fever vaccine has been described in a patient heterozygous for both *CCR5Δ32* and a mutation in a promoter region of the *CCL5* gene [35]. *CCR5Δ32* homozygocity has been linked to the increased susceptibility to West Nile virus (WNV), which is a neurotropic flavivirus related to TBEV and responsible for a similarly wide spectrum of clinical presentations, from asymptomatic through mild febrile disease to severe encephalitis [36, 37]. Epidemiologic studies have shown an increased risk of the clinically overt disease in *CCR5Δ32* homozygotes infected with WNV, which has been hypothesized to result from the impaired lymphocyte influx into csf [38–40]. This findings prompted studies on *CCR5Δ32* distribution in TBE patients, which have shown the increased risk of TBEV meningitis/meningoencephalitis in *CCR5Δ32* bearers [41, 42]. However, the majority of patients with TBE are the wild type allele homozygotes, pointing to a role of additional host factors in defining the susceptibility to TBEV. These factors, for example, individually variable expression of inflammatory and immunoregulatory cytokines, might act either independently of CCR5 pathway or by influencing the CCR5 expression. The latter is highly variable in peripheral blood T lymphocytes in *CCR5 wt/wt* homozygotes and probably determined in a multi-factorial way [25]. One of the proposed mechanisms influencing an individual level of CCR5 expression is its down-regulation by its ligands, described in vitro for CCL5 [43] and as a correlation in vivo for CCL3 [44].

To clarify the role of CCR5 expression and *CCR5* genotype in TBE, we have attempted to evaluate (1) if CCR5 expression in the activated Th lymphocyte population is altered in TBE and/or associated with its clinical presentation, (2) if there is a difference in the baseline expression of CCR5 in TBE patients and the general population, (3) how CCR5 expression in TBE is related to the concentrations of its ligand chemokines, and (4) how the CCR5 and its ligands expression in TBE associate with the *CCR5Δ32* allele.

## Methods

### Patients

The patients hospitalized in the Department of Infectious Diseases and Neuroinfections of the Medical University in Białystok with the serologically confirmed TBEV infection were enrolled into the study and evaluated on admission (examination I), in the early convalescent period prior to discharge 12–16 days later (examination II) and during control visit 5–6 weeks after the admission (examination III). The blood and csf samples for the purpose of the study were obtained together with the material drawn for the diagnostic purposes at these time points, if and when indicated clinically, because of that, not all the samples were available from all the patients.

Anti-TBEV IgM antibodies were detected with Enzygnost Anti-TBE/FSME IgM kit from Siemens (Munich, Germany), following the standard procedure. All the patients included in the study group were either IgM-seropositive on admission or seroconverted by the time of the discharge from hospital.

The group consisted of 76 patients: 6 with meningoencephalomyelitis, 33 with meningoencephalitis, 34 with uncomplicated meningitis, and 3 with a flu-like infection. Of patients with meningoencephalitis or meningoencephalomyelitis, 15 had mild presentation with isolated minor neurologic abnormalities (paresthesia, tremor, pathologic reflexes) and 24 had moderate to severe presentation with altered mental status, paresis and/or multiple focal neurologic symptoms. Of these, blood samples were available for CCR5 cytometric study from 36 patients in examination I, 42 in examination II, and 25 in examination III, and csf samples from 25, 16, and 6 patients, respectively.

Additionally, blood samples from 3 patients hospitalized because of the acute febrile infection, who developed TBEV encephalitis within following 2 weeks, were studied for CCR5 expression and retrospectively classified as obtained during the first (peripheral) phase of TBE (before the examination I time point), but they were not included in the main study group and statistical analysis.

The control blood samples were obtained from (1) healthy blood donors (*n* = 18); (2) patients hospitalized because of acute febrile infections, without meningitis (*n* = 7); and (3) patients hospitalized because of a headache of non-infectious etiology (*n* = 9). To put csf findings in context, CCR5 expression was studied in a group of patients with meningitis of non-TBEV etiology at analogous time-points as in TBE patients: 15 patients with early neuroborreliosis (paired csf available from 7 in examination I, 4 in II, and 6 in III) and 7 patients with non-TBEV aseptic meningitis (including csf samples from 3 in examination I and follow-up examination II and III samples in one of them).

The pilot analysis of lymphocyte fractions was performed in a subset of TBE samples, including blood obtained on admission from 31 patients and csf from 9 patients (6 with meningitis and 3 with severe meningoencephalitis, 5 of them re-evaluated at examination II and 4 at examination III), as well as in a blood of a majority of hospitalized control subjects.

Genotyping was performed in 47 of TBE patients in whom any CCR5 cytometric data were available and in 15 of the healthy controls, as a part of a larger genotyping study conducted simultaneously in our center.

The concentrations of CCL3, CCL4, and CCL5 were measured in serum and csf of a pilot subgroup of patients studied cytometrically: 11 with TBE, 4 with neuroborreliosis, 4 with other lymphocytic meningitis, and 5 in controls without active infection. Following that, additional 45 patients were studied for CCL5 concentration in csf on admission and before discharge (in this group, we have included five additional TBE patients with *CCR5Δ32/wt* genotype not originally enrolled in the cytometric study). Of the total group of 56 TBEV-infected patients in whom CCL5 csf concentration was studied, 1 had flu-like infection, 22 meningitis, 29 meningoencephalitis (13 mild and 16 moderate to severe), and 4 meningoencephalomyelitis. Forty-two of them were genotyped, 32 were *wt/wt* homozygotes and 10 *CCR5Δ32/wt* heterozygotes.

The participants gave informed consent for entry into the study, which was approved by the Ethics Committee of the Medical University in Białystok (approval number R-I-002/372/2011).

### Laboratory examinations

Basic inflammatory parameters intrathecally (total leukocyte and lymphocyte csf count, csf total protein and albumin concentration) and in the periphery (leukocytosis, lymphocytosis) were measured with standard laboratory techniques.

**Fig. 1:**
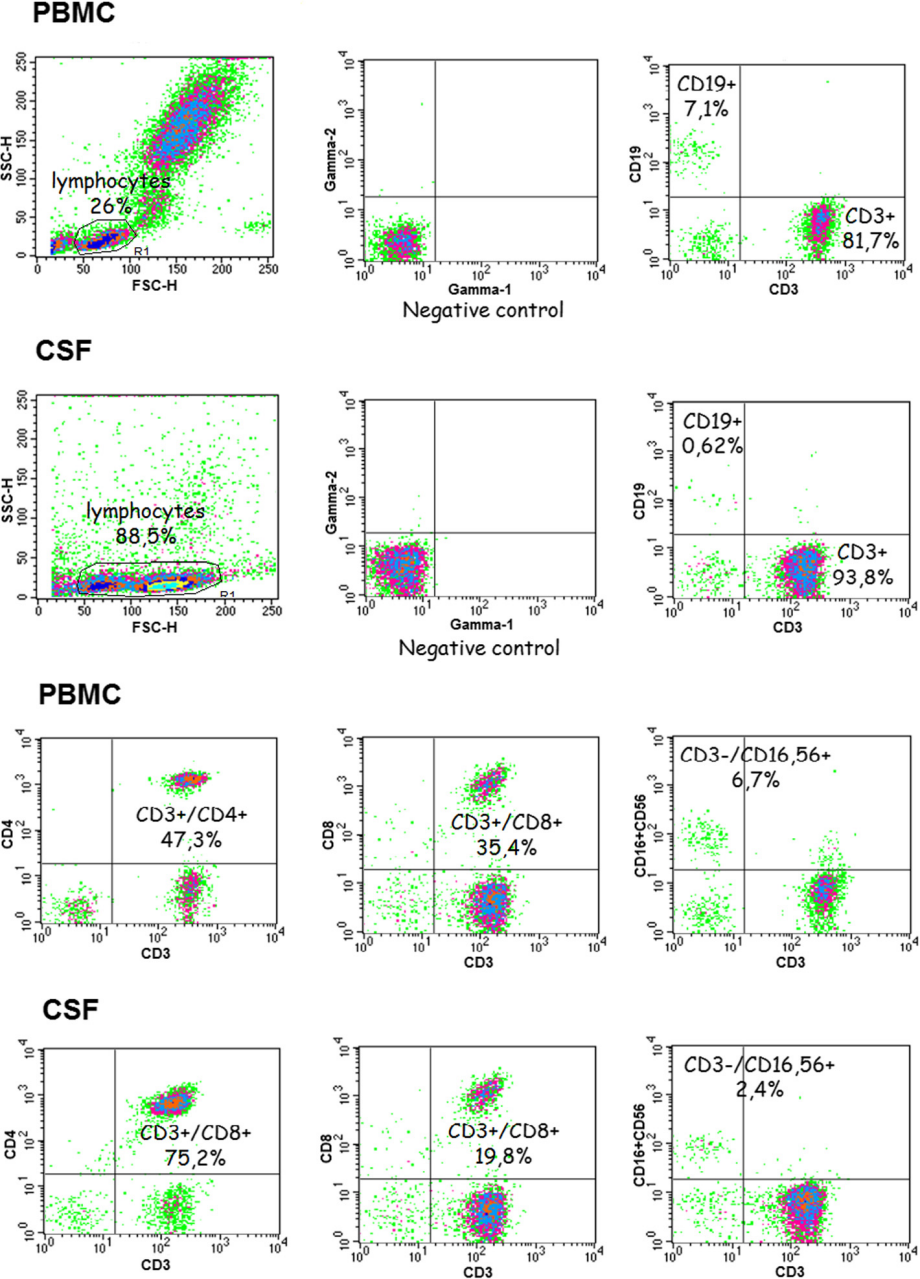
The lymphocyte populations in blood and cerebrospinal fluid of a TBE patient. The representative paired peripheral blood (PBMC—rows 1 and 3 from top) and csf (rows 2 and 4) samples collected from a TBEV-infected patient on admission to hospital were analyzed with Simultest IMK-Lymphocyte, as described in “Methods.” From the left to right in the upper two rows: the gated lymphocyte population; the negative control; identification of B (CD19+) and T (CD3+) lymphocytes; in the lower two rows: T CD3 + CD4+ lymphocytes; T CD3+CD8+ lymphocytes; and NK cells (CD3-CD16+CD56+). The csf lymphocyte population is enriched in CD3+CD4+ cells in comparison with the peripheral blood, at the expense of the other lymphocyte fractions, especially B lymphocytes

The 1 ml samples of the material for flow cytometry were collected into EDTA-coated tubes (blood) and sterile plastic tubes (csf) and analyzed within 6 h on FACSCalibur cytometer. Each time, 20 μl of each monoclonal antibody solution was added to 100 μl of whole blood and incubated for 30 min at room temperature in dark. The blood samples were then treated with lysing solution to lyse erythrocytes and both blood and csf samples were washed with PBS directly before flow cytometry. Simultest™ IMK-Lymphocyte kit from BD Biosciences (San Jose, California, USA) was used to study peripheral blood and csf lymphocyte fractions, strictly following the manufacturer’s instructions. The test is based on a flow cytometry principle and uses pairs of murine monoclonal antibodies (one conjugated with FITC and the other with PE) to measure the fraction of total T lymphocytes (CD3+), T helper/inducer lymphocytes (CD3+CD4+), T suppressor/cytotoxic lymphocytes (CD3+CD8+), B lymphocytes (CD19+), and natural killer (NK) lymphocytes (CD3-CD16 and/or CD56+). The read-outs were analyzed with Simultest IMK-Lymphocyte software. The results in a representative blood and csf paired samples are presented in Fig. 1.

**Fig. 2:**
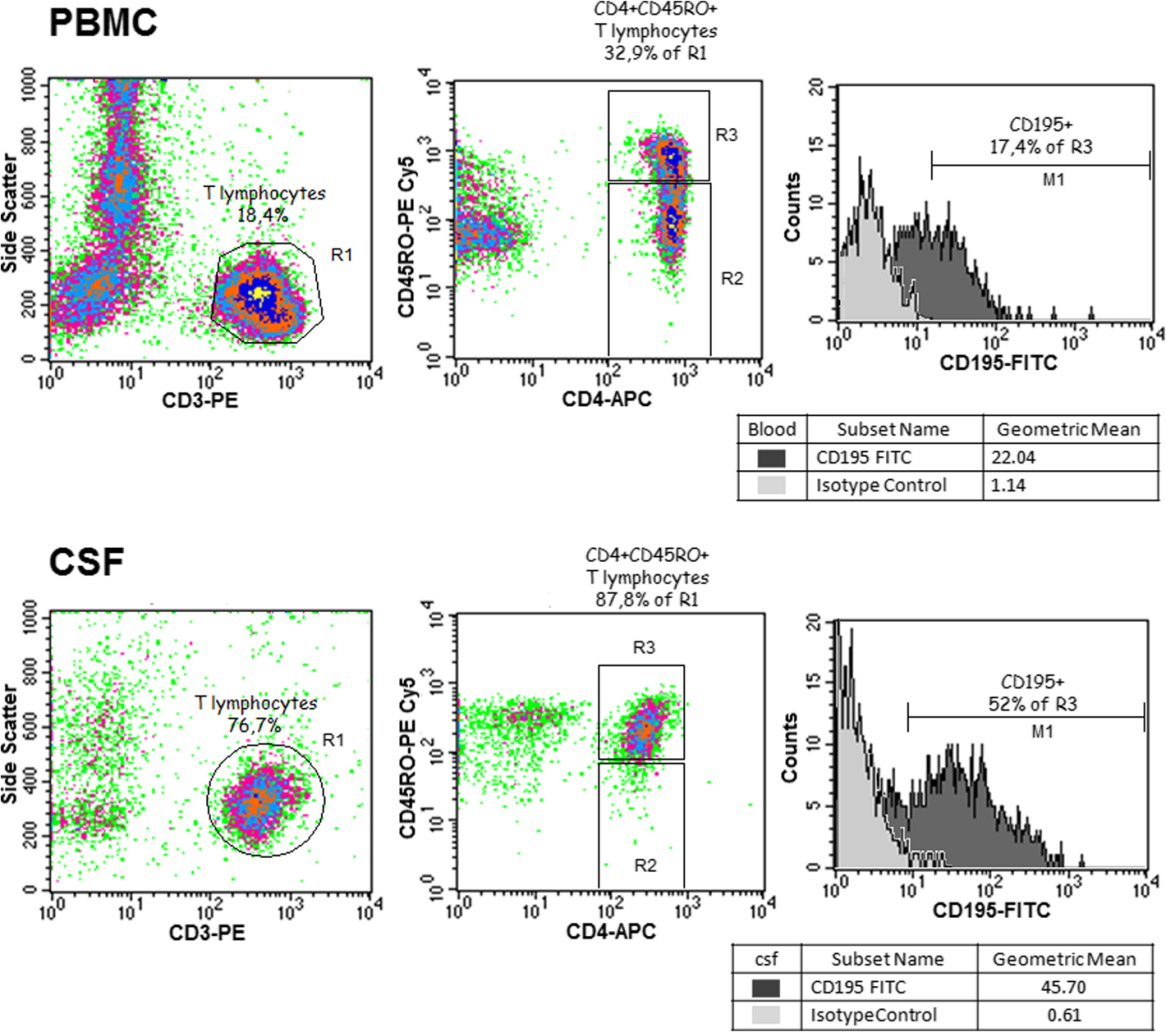
CCR5 expression in blood and cerebrospinal fluid Th lymphocytes from a TBE patient. The representative FACS plots demonstrating the gating strategy and the measured CCR5 (CD195) expression in CD3+CD4+CD45RO+ lymphocyte population in blood (peripheral blood mononuclear cells—PBMC, *upper panel*) and in cerebrospinal fluid (csf, *lower panel*) obtained on admission to hospital from a TBEV-infected patient. Left: T lymphocytes were gated based on CD3 and side scatter dot plot (gate R1). Middle: CD4+CD45RO+ T lymphocytes were gated in selected R1 population based on CD4 and CD45RO dot plot (gate R3). Isotype controls from the same blood and csf samples were used to define the CD45RO+ population. Gate R2 contains CD4+CD45-negative T lymphocytes. Right: a histogram of CD195 expression intensity (*dark gray*) measured on gate R3 (*light gray*—isotype control). The tendency for a preferential migration of T lymphocytes to csf (*left*), for the enrichment of the T lymphocyte population that has migrated into csf in CD4+CD45RO+ lymphocytes (*center*) and for the increased CCR5 expression in the csf CD4+CD45RO+ cells (*right*) is visible

For CCR5 evaluation, the mouse anti-human monoclonal antibodies: PE-labeled anti-CD3 IgG2aκ (clone HIT3a), APC-labeled anti-CD4 IgG1κ (clone RPA-T4), PE-Cy™5 anti-CD45RO IgG2aκ (clone UCHL1), and FITC-labeled anti-CD195 (CCR5) IgG2aκ (clone 2D7/CCR5) were used. PE-Cy™5-conjugated mouse IgG2aκ isotype control was used to assure correct gating of CD45RO+ cells, and the cutoff level for CCR5 expression was determined with FITC-stained mouse IgG2aκ isotype control. Eventually, CCR5 expression was measured in the gated population of CD3+CD4+CD45RO+ cells and expressed as a mean fluorescence index (MFI), calculated from the fraction of CCR5-positive cells and geometric mean of CCR5 fluorescence in CCR5+ population. Data acquisition was performed with CellQuest software (Becton Dickinson). The detailed gating strategy and results in a representative patient are shown in Fig. 2.

QIAamp DNA Blood Mini Kit (QIAgen, Hilden, Germany) was used to extract genomic DNA from the blood samples collected to EDTA tubes, following the manufacturer’s protocol. DNA was re-suspended in 200 μL of AE buffer (QIAgen, Hilden, Germany) and stored at 4 °C for further analyses. To analyze *CCR5Δ32* variation, PCR with sequence-specific primers was used as described previously [45]. Visualization under UV light was performed after electrophoresis on the 2.5 % agarose gel (SIGMA, Saint Louis, USA) stained with DNA-star dye (Lonza Inc., Rockland, USA).

Chemokine concentrations were measured in serum samples obtained from 5 ml of blood collected for clotting and centrifuged within an hour after collection and in csf samples obtained on the same day. All the materials were frozen on the day of collection, kept at −70 °C and thawed directly before performing examinations. CCL3, CCL4, and CCL5 concentrations were measured with commercial ELISA kits from R&D Systems, Inc. (Minneapolis, MN, USA), strictly following the manufacturer’s instructions. The minimum detectable concentrations were 10.0 pg/ml for CCL3, 4.0 pg/ml for CCL4, and 2.0 pg/ml for CCL5; read-out below the detection limit were considered zero.

### Statistical analysis

The statistical analysis was performed with STATISTICA 10 software with non-parametric tests: Kruskal-Wallis ANOVA and Mann-Whitney *U* test for independent, Friedmann ANOVA and Wilcoxon pair tests for dependent variables. CCR5 expression and chemokine concentrations were compared between the study groups and analyzed with the respect to the clinical manifestation (meningitis, meningoencephalitis, memingoencephalomyelitis), severity (mild versus moderate/severe cns involvement, presence versus lack of altered consciousness), clinical course (typical biphasic with a distinct flu-like phase of peripheral infection versus monophasic), and *CCR5Δ32* genotype in TBE group. Correlation between CCR5 expression and CCL5 concentration, as well as between these variables and basic laboratory parameters, were assessed with the R Spearman test. *p <* 0.05 was considered statistically significant.

## Results

### Peripheral blood cytometry

On admission, TBE patients had higher median leukocytosis (9.250/μl) and lower lymphocyte count (1.295/μl) than other study group, which normalized to 6.875/μl and 2.076/μl, respectively, in examination II before discharge. The TBE peripheral blood lymphocyte population contained 68 % of T CD3+ cells (890/μl), including 33 % of CD4+ (519/μl) and 24 % of CD8+ lymphocytes (334/μl) and 16 % of B cells (225/μl) and 13 % of NK cells (178/μl) on admission. Both the proportion and absolute number of CD3+ and CD3+CD4+ tended to be lower in TBE than in other study groups but increased to 74 % (1289/μl) and 43 % (889/μl), respectively, before discharge, undistinguishable from the other subjects.

Of the gated CD3+CD4+ lymphocytes, a median of 42 % was of an activated/memory subtype, which was stable in examinations I, II, and III and did not differ from other study groups. The CCR5 expression did not differ between the TBE patients and other groups, including healthy controls, did not differ between the subgroups of TBE patients, and did not change between examinations I, II, and III, but was highly individually variable with values in examinations I and II in the same patients correlated (correlation strength 0.6, *p <* 0.05). In three patients studied in the first phase of the disease and three patients with flu-like TBEV infection, the CCR5 expression fits in the range of values found in the neurologic phase.

### Cerebrospinal fluid cytometry

Basic csf inflammatory parameters in TBE, neuroborreliosis, and other meningitis patients are summed up in Table 1. On admission, TBE patients had lower pleocytosis and especially the lower csf lymphocyte count in comparison with neuroborreliosis and other lymphocytic meningitis patients. While there was a general tendency for a simultaneous normalization of the csf inflammatory parameters in all the neuroinfection groups, the lymphocyte count in TBE was constant between examinations I and II and as a result, the lymphocyte contribution to pleocytosis tended to increase from slightly over 50 % on admission to >95 % in almost all the convalescent samples. There was a higher pleocytosis in TBE meningoencephalomyelitis than in other TBE presentations and an analogous tendency for the lymphocytosis.

**Table 1:** The median values of the basic cerebrospinal fluid parameters in the patient groups studied cytometrically for CCR5 expression

	Pleocytosis/μl			Lymphocyte count/μl			Protein (mg/dl)^d^			Albumin (mg/dl)^e^		
	I	II	III	I	II	III	I	II	III	I	II	III
TBE total (*n* = 76)^f^	73^a^	43^c^	17^c^	39^a^	42	16^c^	56	58	43^c^	41	39	29^c^
TBE—M (*n* = 34)^f^	79	52	18^c^	44	47	18^c^	55	59	42^c^	43	42	30^c^
TBE—ME (*n* = 33)^f^	64	41	13^c^	37	34	14^c^	57	55	42^c^	40	37	27^c^
TBE—MEM (*n* = 6)^f^	171^b^	38	26	77	30	23	74	86	59	52	41	39
NB (*n* = 15)^f^	234^a^	52^c^	21^c^	207^a^	50^c^	18^c^	119	60^c^	44^c^	77	46^c^	33^c^
AM (*n* = 7)^f^	213^a^	22^c^	15	102^a^	20^c^	33	60	44^c^	43	41	33^c^	22

*TBE* tick-borne encephalitis, *M* meningitis, *ME* meningoencephalitis, *MEM* meningoencephalomyelitis, *NB* neuroborreliosis, *AM* aseptic meningitis (non-TBE, non-NB)

I—examination on admission to hospital; II—10–14 days later, III—6–8 weeks later

^a^significant difference between TBE and other neuroinfection groups (*p* < 0.05)

^b^significant difference in comparison with TBE-M and TBE-ME groups

^c^significant decrease in comparison with examination I (*p* < 0.05)

^d^the upper limit of normal is 45 mg/dl

^e^the upper limit of normal is 30 mg/dl

^f^the group size in examination I; in examinations II and III some patients from each group were not available for study

The lymphocyte subpopulations in csf of nine TBE patients on admission are shown in Table 2; repeated examination in the same patients in the convalescent period gave essentially the same results (not shown). There was a higher CD8+ cell percentage in the csf from patients with severe meningoencephalitis than with meningitis (*p <* 0.05) and in patients with altered consciousness than in those with normal mental status (*p* = 0.055). Activated CD45RO+ cells constituted a median of 75 % of csf Th lymphocyte population in examination I, 56 % in examination II, and 71 % in examination III (36 cells/μl, 21/μl ,and 11/μl, respectively), independent of the clinical manifestation. In comparison with circulating lymphocytes, csf lymphocyte population of the TBE patients was significantly enriched in T CD3+ and Th CD3+CD4+ and especially in activated Th CD3+CD4+CD45RO cells at all three examination time-points (Figs. 1 and 2).

**Table 2:** The lymphocyte subpopulations in the cerebrospinal fluid obtained on admission to hospital from a subset of tick-borne encephalitis (TBE) patient group, assessed as described in “Methods.” The results are shown as a median fraction of the lymphoid cell population and as a median absolute cell number per microliter calculated from the total lymphocyte count (in parentheses)

Patient group	Cell population				
	T CD3+	Th CD3+CD4+	Tc CD3+CD8+	B	NK
TBE total (*n* = 9)	95 % (42)	79 % (30)	16 % (10)	0.4 % (<1)	3 % (3)
TBE—M (*n* = 6)	95 % (50)	81 % (43)	15 %^a^ (8)	0.5 % (1)	4 % (3)
TBE—ME (*n* = 3)	96 % (42)	64 % (29)	28 %^a^ (11)	0.3 % (<1)	2 % (1)

*TBE* tick-borne encephalitis, *M* meningitis, *ME* meningoencephalitis

^a^ *p* < 0.05 for the difference between M and ME patients

**Fig. 3:**
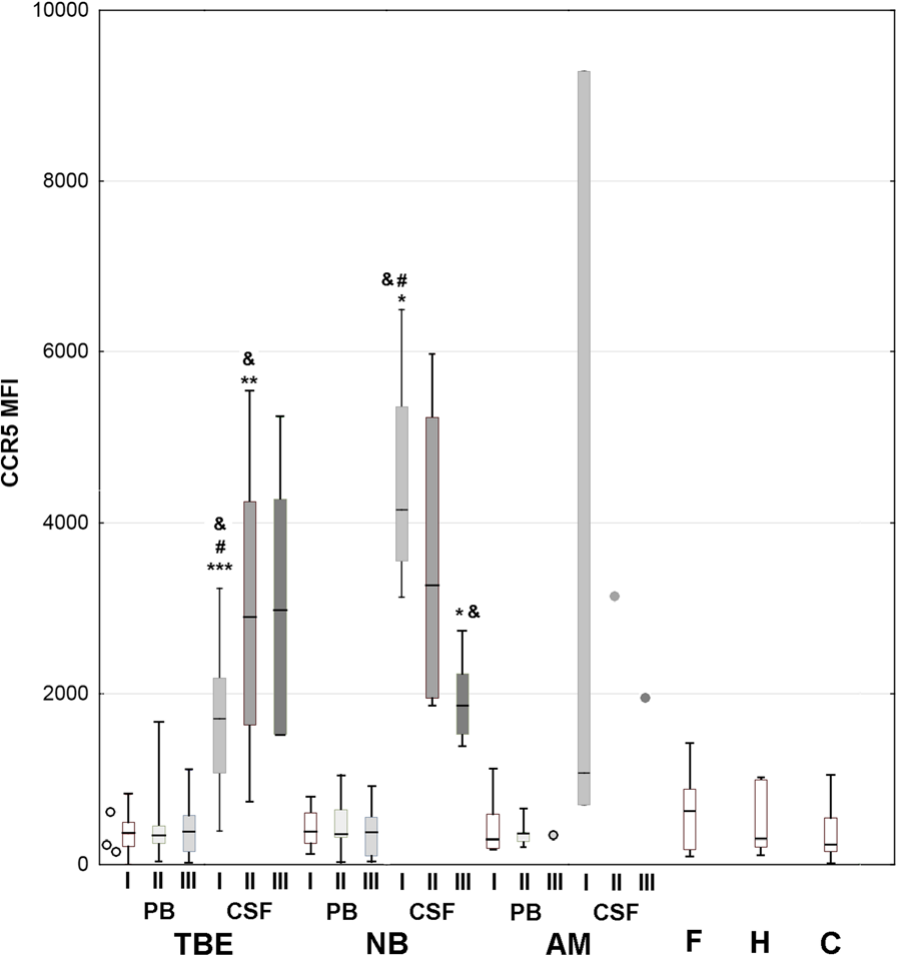
CCR5 expression in TBEV-infected patients compared to other patient groups and healthy controls. Expression of CCR5 in the activated Th lymphocyte population (CD3+CD4+CD45RO) measured cytometrically as described in “Methods” and expressed as mean fluorescence index (MFI). Comparison of values in peripheral blood (PB) and cerebrospinal fluid (CSF) of patients with tick-borne encephalitis (TBE), early cns neuroborreliosis (NB), and other aseptic meningitis (AM) on admission to hospital (I), 10–14 days later (II) and 4–6 weeks later (III), as well as in peripheral blood of subjects without meningitis/encephalitis: with acute febrile infection without cns involvement (AF), with headache of non-infectious etiology (H) (both groups—at admission to hospital), and in healthy blood donors (C). Shown are median (*horizontal line*), quadrilles (*box*), and minimum and maximum values (whiskers); when only individual values are available in AM csf, they are presented as *circles*; the *empty circles* to the left denote blood samples from three patients studied in the peripheral phase of TBE, up to 2 weeks between the onset of meningitis (not included in the statistical analysis). There was no difference between the study groups and examination time-points in the peripheral blood expression. *significant difference between paired blood and csf examinations, *p* < 0.05; **significant difference between paired blood and csf examinations, *p* < 0.01; ***significant difference between paired blood and csf examinations, *p* < 0.001; ^#^significant difference between TBE and NB csf, *p* < 0.05; &significant difference between the examination on admission and during folow-up, *p* < 0.05

CCR5 was expressed by a median 39 % of Th CD3+CD4+CD45RO lymphocytes in the TBE csf on admission—an over twofold and highly significant enrichment in comparison with the peripheral blood lymphocyte population. There was a further increase in CCR5-positive fraction in examination II (48 %) sustained in examination III (57 %). As a result, the absolute number of CCR5-positive activated Th lymphocytes in csf was relatively constant in spite of a decreasing total pleocytosis: 12 cell/μl in examination I, 14/μl in examination II, and 6/μl in examination III, when this fraction constituted almost 40 % of the total csf leukocyte population. The intensity of staining of CCR5-positive cells tended to be higher in csf than in peripheral blood, resulting in several-fold increased MFI (Figs. 2 and 3). CCR5 csf expression tended to be similarly increased in a small group of patients with aseptic meningitis, while in neuroborreliosis, it was even higher: 52 % of activated Th lymphocytes was CCR5-positive, in combination with higher csf lymphocyte count resulting in 10 times higher absolute number of activated CCR5+ cells than in TBE.

**Fig. 4:**
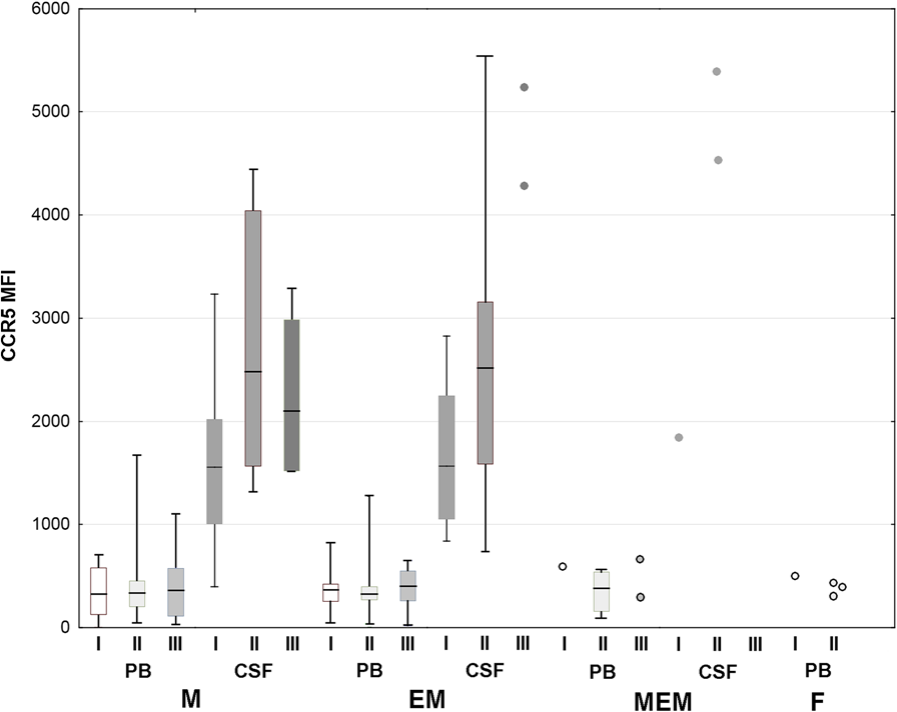
CCR5 expression in TBEV-infected patients dependent on the clinical form of the disease. Expression of CCR5 in the activated Th lymphocyte population (CD3+CD4+CD45RO) measured cytometrically as described in “Methods” and expressed as mean fluorescence index (MFI), in peripheral blood (PB) and cerebrospinal fluid (CSF) of patients with the infection with tick-borne encephalitis virus (TBEV) presenting as meningitis (M), meningoencephalitis (ME), and meningoencephalomyelitis (MEM) as well as in peripheral blood of patients with a mild flu-like TBEV infection (F) on admission to hospital (I), 10–14 days later (II) and 4–6 weeks later (III). Shown are median (*horizontal line*), quadrilles (*box*), and minimum and maximum values (*whiskers*); in the least numerous groups, not allowing for statistical analysis, only individual values are shown as *circles* instead. There was no significant difference between the CCR5 expression in patients with different clinical presentation of TBEV infection

Within TBE group, csf CCR5 expression tended to be relatively high in patients with meningoencephalomyelitis, but the number of csf samples available for cytometry was too small for a statistical confirmation. There was no difference between meningitis and meningoencephalitis groups (Fig. 4). CCR5 expression did not differ between patients with mild and moderate/severe TBE meningoencephalitis, between patients with monophasic versus biphasic course of the disease, and patients with normal versus altered mental status.

CCR5 csf expression on admission was significantly (*p <* 0.05) higher in men than in women in TBE group.

### Genotyping

There were no *CCR5Δ32* homozygotes identified in the study groups. Five *CCR5Δ32/wt* heterozygotes were detected among the healthy controls and 7 among genotyped TBE patients. Unfortunately, limited material was available for cytometry from TBE *CCR5Δ32/wt* heterozygotes, including paired blood and csf samples from 3 patients (in individual patients: from examinations I and II, only examination I, only examination II) and only examination II–III blood samples from the remaining four.

**Fig. 5:**
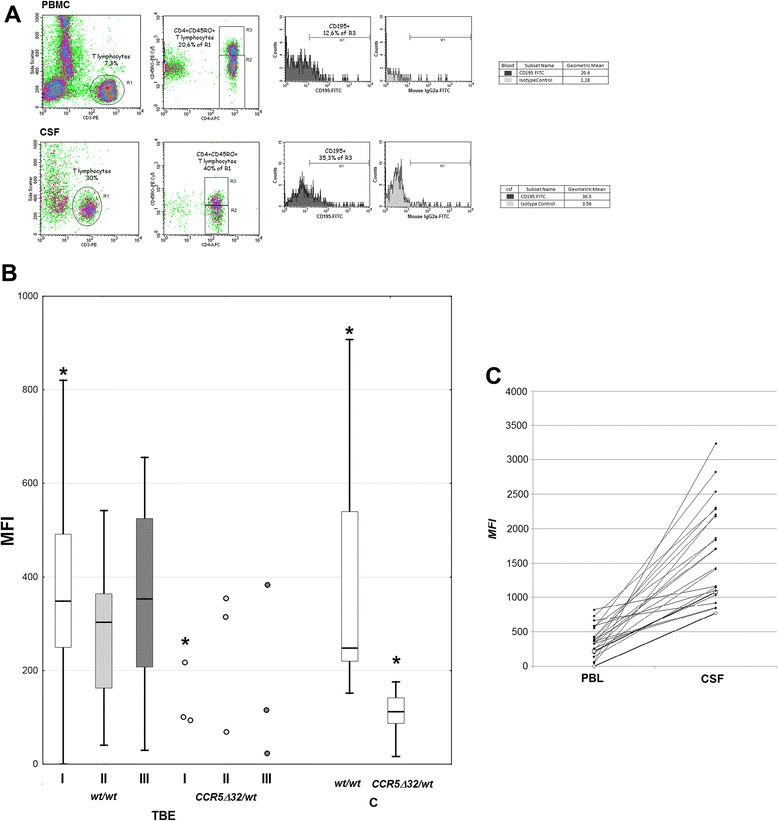
CCR5 expression in a TBEV-infected patients with *CCR5Δ32/wt* versus *wt/wt* genotype. **a** FACS plots of peripheral blood (PBMC, *upper panel*) and cerebrospinal fluid (csf, *lower panel*) obtained on admission to hospital from a TBE patient with *CCR5Δ32/wt* genotype. Left: T lymphocytes were gated based on CD3 and side scatter dot plot (gate R1). Middle: CD4+CD45RO+ T lymphocytes were gated in selected R1 population based on CD4 and CD45RO dot plot (gate R3). Isotype controls from the same blood and csf samples were used to define the CD45RO+ population. Gate R2 contains CD4+CD45-negative T lymphocytes. Right: a histogram of CD195 expression intensity (*dark gray*) measured on gate R3 (*light gray*—isotype control). **b** CCR5 expression in the activated Th lymphocytes population in the peripheral blood in TBEV-infected patients stratified by *CCR5* genotype, measured as in A and expressed as MFI, on admission (I), 10–14 days later (II) and 4–6 weeks later (III). Data from *wt/wt* homozygotes (*n* = 20 in I, 25 in II, and 17 in III) and from *CCR5Δ32/wt* heterozygotes (*n* = 3 at each time point) compared with healthy controls (C, *n* = 10 for *wt/wt* and 5 for *CCR5Δ32/wt*). Shown are median (*horizontal line*), quadrilles (box), and minimum and maximum values (*whiskers*); in TBE *CCR5Δ32/wt* groups, individual values are shown as *circles*. *significant difference between genotypes with *p* < 0.05; the same results were obtained when CCR5-positive cell fraction was analyzed instead of MFI as a measure of CCR5 expression; **c** CCR5 expression (presented as MFI) in paired csf (CSF) and peripheral blood lymphocyte (PBL) populations from TBEV-infected patients on admission to hospital; values from *wt/wt* homozygotes are shown as filled diamonds and from two *CCR5Δ32/wt* heterozygotes as empty symbols; the CCR5 expression in *CCR5Δ32/wt* patients is low, but there is an apparent enrichment of the csf population in CCR5 expressing cells, analogously to *wt/wt* homozygotes

**Fig. 6:**
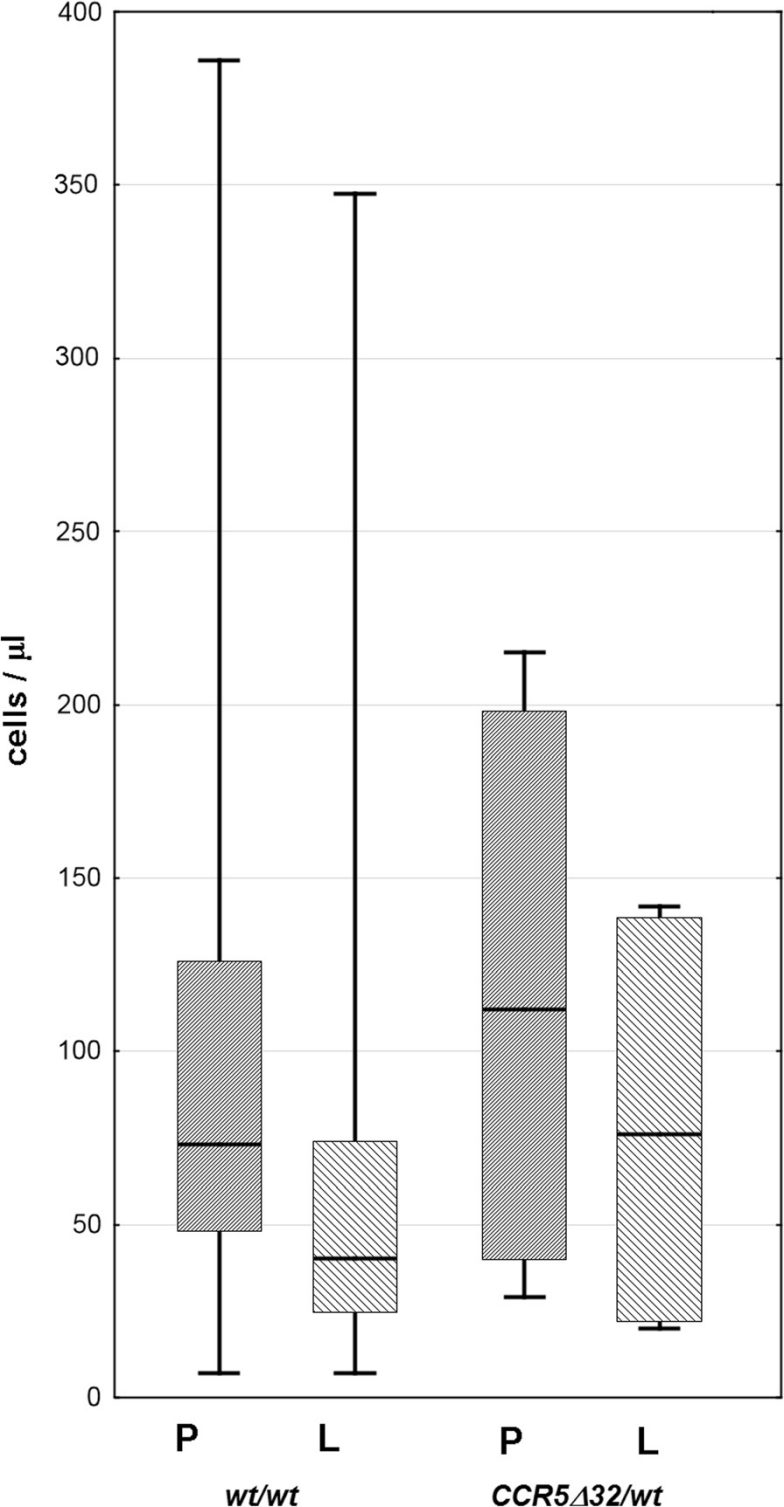
The cerebrospinal fluid cytosis in TBEV-infected patients stratified by *CCR5* genotype. The total pleocytosis (P) and csf lymphocyte count (L) on admission to hospital in TBEV-infected patients with *wt/wt* (*n* = 39) and *CCR5Δ32/wt* (*n* = 7) genotype (data from a single *wt/wt* patient with an extremely high initial pleocytosis of 1196 cells/μl are not shown for clarity). Shown are median (*horizontal line*), quadrilles (*box*), and minimum and maximum values (*whiskers*). There was no significant difference between the patients with different genotypes

The representative cytometric plots of CCR5 expression in CD3+CD4+ CD45RO cells in *CCR5Δ32/wt* heterozygote are shown in Fig. 5a. The CCR5 expression in the peripheral blood was lower in *CCR5Δ32* carriers than in *wt/wt* homozygotes both in the terms of the fraction of CCR5-positive cells and CCR5 MFI, but the expression levels associated with both genotypes overlapped, especially in TBE patients in examinations II and III (Fig. 5b). In csf samples from *CCR5Δ32* heterozygotes, CCR5 expression tended to be at the lower end of values found in *wt/wt* genotype, but the enrichment in CCR5+ cells was evident. Csf-activated Th population contained from 17 to 41 % of CCR5-positive cells, overlapping with values from *wt/w*t heterozygotes (Fig. 5c). Total lymphocytic pleocytosis was not reduced in *CCR5Δ32* bearers (Fig. 6), and the Th lymphocyte population tended to be enriched in activated cells analogously to the rest of TBE patients. The total protein and albumin csf concentration did not differ between *CCR5Δ32/wt* and *wt/wt* patients (not shown). Five of *CCR5Δ32/wt* patients presented with meningoencephalitis of mild to moderate severity and only two with meningitis, suggesting a tendency for a more severe manifestation in the presence of *CCR5Δ32*, but the trend was not statistically significant.

### Chemokine expression

In the pilot analysis, serum concentrations of CCL3, CCL4, and CCL5 did not differ between controls and patients with different forms of lymphocytic meningitis, including TBE. Concentrations of CCL3 and CCL4 in csf were not increased in any of the meningitis/encephalitis groups in comparison with controls and were not higher in csf than in serum, clearly not forming a chemotactic gradient towards cns.

**Fig. 7:**
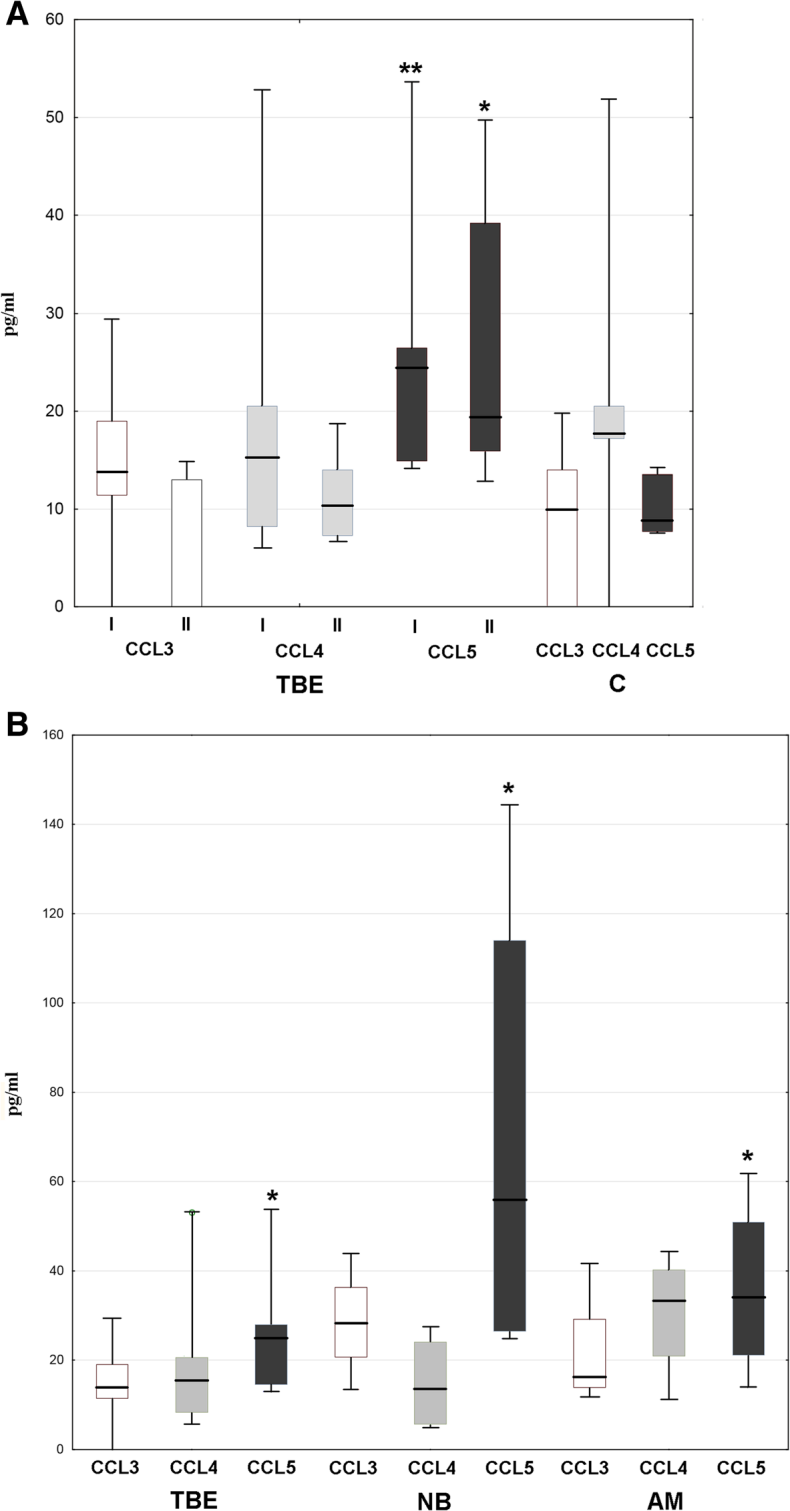
Concentrations of CCR5 ligands in cerebrospinal fluid of TBEV-infected patients. Concentrations of CCL3, CCL4, and CCL5 in the cerebrospinal fluid of a pilot group of TBE patients on admission to hospital (I, *n* = 11) and in the early convalescent period after 10–14 days (II, *n* = 6) measured immunoenzymatically as described in “Methods” and expressed in pg/ml. The detection limit was 10 pg/ml for CCL3, 4 pg/ml for CCL4, and 2 pg/ml for CCL5. Shown are median (*horizontal line*), quadrilles (*box*), and minimum and maximum values (*whiskers*). **a** Elevated concentration of CCL5, but not CCL3 and CCL4, in comparison with controls with no infectious nor inflammatory pathology (C, *n* = 5); *significant difference in comparison with C (*p* << 0.05); **significant difference in comparison with C, *p* < 0.01; **b** Concentration of CCL5 in TBE on admission compared to neuroborreliosis (NB, *n* = 4) and aseptic meningitis (AM, *n* = 4) patients—no statistically significant difference between the groups, *significantly higher median concentration in comparison with controls

The csf concentration of CCL5 was significantly elevated in TBE patients on admission and remained elevated in examination II, although with a tendency to decrease (Fig. 7a). The same tendency was present in the groups of neuroborreliosis and the other aseptic meningitis patients. In neuroborreliosis, the median CCL5 concentration tended to be threefold higher than in TBE, although the difference did not reach the level of statistical significance (Fig. 7b). CCL5 serum concentrations in both TBE patients and controls were extremely high, which is in accordance with the literature data and reflects a thrombocyte interference during the serum isolation and handling and not the situation in vivo [46]. As a result, CCL5 gradient between csf and serum could not be evaluated.

**Fig. 8:**
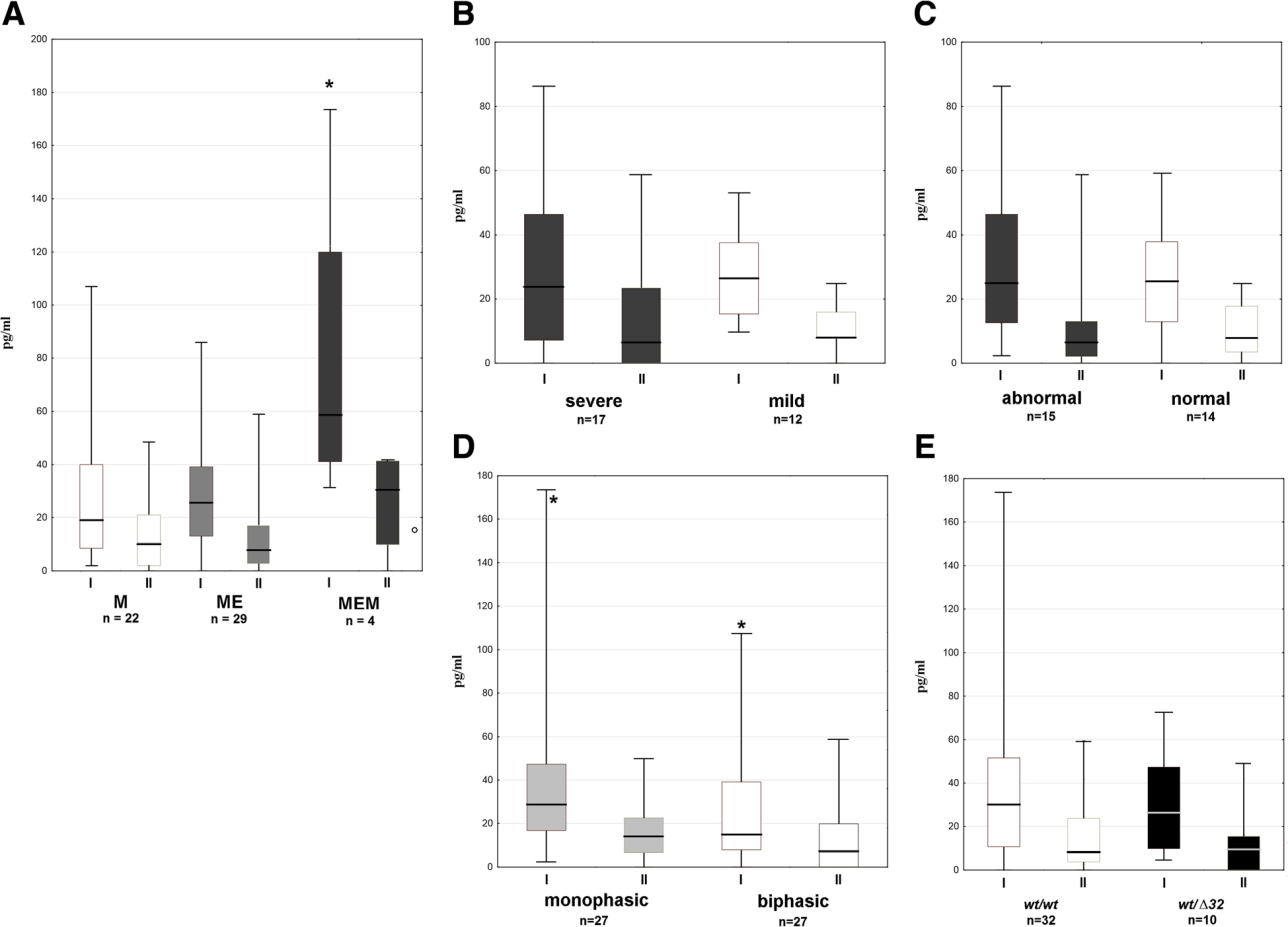
Variability of CCL5 concentration in cerebrospinal fluid of TBEV-infected patients. Elevated concentration of CCL5 in the cerebrospinal fluid of a group of TBE patients on admission to hospital (I, *n* = 56) and in the early convalescent period after 10–14 days (II, *n* = 47) measured immunoenzymatically as described in “Methods,” in picograms per milliliter (pg/ml). Lower detection limit was 2 pg/ml. Shown median (*horizontal line*), quadrilles (*box*), and extreme values (*whiskers*). **a** Patients stratified dependent on the clinical manifestation of TBEV infection: meningitis (M, *n* = 22), meningoencephalitis (ME, *n* = 29), meningoencephalomyelitis (MEM, *n* = 4); *significantly higher in MEM comparison with the two other groups (*p* < 0.05); a value in a single patient with a flu-like infection is shown to the right as an *empty circle*; **b** Patients with meningoencephalitis stratified dependent on the clinical severity: mild (*n* = 12), moderate to severe (with paresis, multifocal neurologic signs, and/or altered consciousness; *n* = 17); **c** Patients with meningoencephalitis stratified dependent on the mental status: normal (*n* = 14), abnormal (agitation, disorientation, somnolence, *n* = 15); **d** Comparison between the groups of patients with the monophasic (*n* = 27) versus biphasic (*n* = 27) clinical course of the disease, *significant difference with *p* < 0.05; **e** Subgroup of 42 patients with TBEV infection stratified by the *CCR5* genotype: *wt/wt* (*n* = 32) or *CCR5Δ32/wt* (*n* = 10)

Following that, csf CCL5 was studied in a larger group of TBE patients, which confirmed its increased concentration and its decrease before discharge in comparison with the examination on admission (*p* < 0.01) (Fig. 8). Median CCL5 was significantly higher in patients with meningoencephalomyelitis in comparison with meningitis and meningoencephalitis groups. In meningoencephalitis, CCL5 concentration was not different in comparison with the meningitis group and did not depend on the severity of the cns involvement. It was significantly higher in patients with monophasic versus typical biphasic clinical course of the disease. In a patient with a flu-like TBEV infection, CCL5 in the csf was close to the lower end of values found in TBE meningitis/meningoencephalitis but still higher than in any of the control samples. Finally, CCL5 csf concentration did not correlate with *CCR5* genotype.

**Fig. 9:**
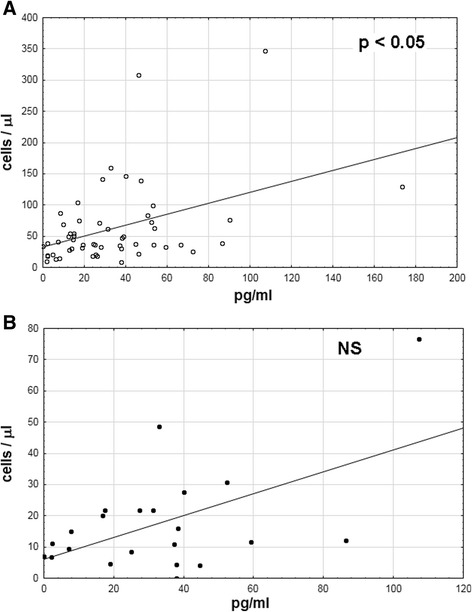
Correlation of the CCL5 concentration with cerebrospinal fluid cytometric parameters. Correlation of the CCL5 concentration in the cerebrospinal fluid (*horizontal axis*, pg/ml) with csf cytometric parameters (*vertical axis*, cell/μl) in TBEV-infected patients, measured and calculated as described in “Methods”; **a** Csf lymphocyte count—significant correlation with a strength of 0.38 and *p* < 0.05; **b** The absolute number of CCR5-positive activated Th lymphocytes (T CD3+CD4+CD45RO CCR5+ cells)—an analogous tendency for a positive correlation as in A, not reaching the level of statistical significance. *NS* not significant

Csf CCL5 concentration correlated significantly with the total pleocytosis, lymphocyte count, total protein, and albumin concentration. There was also a tendency for a positive correlation with a number of CCR5-positive activated Th cells per microliter, although it did not reach the level of statistical significance (Fig. 9).

## Discussion

Although an important role of CCR5 in flavivirus encephalitis has been suggested by both animal models and epidemiologic studies, to our knowledge, its expression has not been analyzed in a patient group with respect to the clinical presentation and genetic background so far. To assess its role in TBE, we have studied CCR5 in activated Th lymphocytes (CD3+CD4+CD45RO+) in csf, a cell population that is relatively accessible, reflects the intrathecal inflammatory/immune response, is important for TBEV clearance from cns, and which appears to be preferentially recruited into csf of TBE patients. Interestingly, in our pilot experiments, we have found a tendency for a higher proportion of CD3+CD8+ versus CD3+CD4+ lymphocytes in csf lymphoid cell population in patients with the more severe manifestation of TBE, which is consistent with the hypothesis that cytotoxic T cells are involved in TBE-related immunopathology while Th cells we chose for the study are protective [13, 18].

CCR5 is commonly expressed by intrathecal Th lymphocytes in both infectious (neuroborreliosis) and non-infectious (multiple sclerosis, MS) cns inflammation, usually alongside CXCR3 receptor for chemokines CXCL9 and CXCL10, but its role remains debatable [26, 28–30]. In MS, CXCR3 is considered responsible for a lymphocyte migration, while the presence of CCR5 is secondary to its expression on the activated (CD45RO+) lymphocytes preferentially recruited to the inflammatory csf [28, 29]. High CCR5 expression has been observed on csf CD4+ and CD8+ lymphocytes in neuroborreliosis, where it was also co-expressed with CXCR3 [26, 27, 30]. According to Giunti et al., CCR5 does not contribute significantly to lymphocyte migration into csf in neuroborreliosis but may be up-regulated on activated Th lymphocytes intrathecally and facilitate their subsequent migration to the nervous tissue [26]. In viral encephalitis, the role of CXCR3 and CCR5 has been studied mainly in animal models and shows differences depending on virus type and strain virulence [15, 20, 47]. The lack of CCR5 expression has no apparent consequences in mice infected with lymphocytic choriomeningitis virus [23] and paradoxically increases leukocyte influx and intrathecal inflammation in murine model of herpes simplex type 1 encephalitis [48]. On the other hand, CCR5 seems to be functional and active alongside CXCR3 in WNV-encephalitis model [19, 32]. CCR5-negative knock-out mice have a normal inflammatory/immune response in the periphery, but leukocyte migration into cns is impaired, accompanied by an inability to clear virus from the brain tissue and an increased mortality. The expression of other chemokine receptors (CCR1, CCR2, CXCR3) and of chemokines (CXCL9, CXCL10, CCL2, CCL3, CCL4, and CCL5) is increased but is unable to compensate for the lack of CCR5, suggesting that the CCR5 role in the infection with neurotropic flavivirus is non-redundant and distinct from encephalitides of other etiology [32]. However, CXCR3 has been found on csf Th lymphocytes from TBE patients, and its ligand CXCL10 has been detected simultaneously in csf by Lepej et al., so the contribution from this receptor remains probable too [16].

In our group of TBEV-infected patients, an activated Th lymphocyte population in csf was enriched in CCR5-expressing cells, suggesting that either they were preferentially attracted into csf or that, alternatively, CCR5 was up-regulated on activated Th lymphocytes in the pro-inflammatory csf environment, possibly influencing their further trafficking, as suggested by Giunti et al. for neuroborreliosis [26]. Both possibilities are not mutually exclusive and point to CCR5 involvement in TBE pathogenesis. Because of a suspected particular role of CCR5 in flavivirus infections, we expected its expression to be higher in TBE compared to other forms of meningitis. This was, however, not the case as the CCR5 csf expression was higher in neuroborreliosis than in TBE. The feature specific for TBE was a tendency for a continuing increase of CCR5 expression at least between the time of hospital admission and early convalescent period 2 weeks later, contrasting with the decrease in other patient groups. These trends are consistent with a relatively low initial lymphocyte count accompanied by the prolonged inflammatory changes in TBE csf, described in previous studies and observable in our study group as well [49].

Vividly increased expression of CCL5 in brains of mice with WNV-encephalitis and TBE suggests it as a main CCR5 ligand in this setting [32, 50, 51]. Our previous results pointed to a possible role of two of CCR5 ligands, CCL5 (RANTES) and CCL3 (MIP-1α) in TBE pathogenesis, although their chemotactic gradient towards csf could not be proven [52, 53]. In the recent study, Palus et al. found no increased CCL3, CCL4, and CCL5 concentrations in serum of TBE patients, which is in agreement with our current results [54]. On the other hand, we have confirmed the concentration of CCL5, but not CCL3 and CCL4, to be significantly increased in the csf of the pilot group of TBE patients. When subsequently studied in a larger patient group, CCL5 csf concentration was increased in comparison with healthy controls, correlated with the csf inflammatory parameters and tended to correlate with the csf Th CCR5 expression. When we compared CCL5 expression between TBE, neuroborreliosis, and other aseptic meningitis patients, the differences between these groups were analogous to the trends observed for CCR5 expression: in TBE, the initial CCL5 concentration was low in comparison with neuroborreliosis, but its increase was characteristically protracted into the convalescent phase. These data are consistent with CCL5 and CCR5 cooperating in driving lymphocyte migration into csf in TBE and influencing the course of the intrathecal inflammation. CCL5 concentration was relatively high in TBE patients with meningoencephalomyelitis, who also had a tendency for a higher intrathecal CCR5 expression and pleocytosis. The other studies also show the correlation of the intrathecal concentrations of CCR5 ligands with a severity of neurologic involvement. The mouse strains genetically susceptible to TBEV present with a higher expression of CCL3, CCL4, and CCL5 in the brain parenchyma during experimental TBE compared to relatively resistant strains [55], and high CCL5 concentration in patients infected with Japanese encephalitis virus is associated with mortality [56]. Interestingly, in a patient with a flu-like TBEV infection, a concentration of CCL5 in csf was higher than in any of the healthy controls, without any signs of concomitant or subsequent intrathecal inflammation, suggesting CCL5 intrathecal up-regulation even without the clinically overt cns infection.

Another line of evidence supporting the role of CCR5 in flavivirus infection comes from the studies comparing *CCR5* genotypes in patients with healthy subjects. According to Glass et al. and Lim et al., *CCR5Δ32* homozygotes have a several-fold increased risk of a symptomatic WNV infection [37, 38]. The data suggest that almost all infected *CCR5Δ32* homozygotes develop a symptomatic disease versus a minority of the general population [19]. The increased frequency of *CCR5Δ32* allele and *CCR5Δ32/CCR5Δ32* genotype has been detected in Lithuanian patients with TBE [41, 42], but not in patients from Novosibirsk area (Asiatic part of Russia) likely infected with the Siberian TBEV subtype [7, 57]. The discrepancy between the European and Siberian TBE studies could result from the influence of genetic background in distinct human populations or from the differences between two separate TBEV types. The main difference between the results of WNV and European TBEV studies was the susceptibility of *CCR5Δ32/wt* heterozygotes. No association of *CCR5Δ32* heterozogosity with WNV infection was observed, suggesting that even a reduced expression of CCR5 is sufficient for protection [39], but in the European patients, increased TBE risk was associated with *CCR5Δ32* heterozogosity, as if the quantitatively changed CCR5 expression was sufficient to alter susceptibility to TBEV [41]. The reasons for that difference are unclear, especially that mechanisms by which CCR5 deficiency could influence susceptibility to flavivirus in humans has not been studied. The simple explanation in agreement with the animal models is an impaired lymphocyte influx into cns, resulting in a more significant and clinically manifest neuroinfection [39]. However, this would also favor clinically more severe cns involvement, which has not been confirmed [38, 39, 42]. In the contrary, a large epidemiologic study of blood donors has shown that the *CCR5Δ32* correlates with the risk of any clinically overt WNV-related disease (including mild flu-like infections) versus asymptomatic infection and not specifically with meningitis/encephalitis [40]. That suggests that *CCR5Δ32* associates with the impairment of the early peripheral response to WNV and TBEV resulting in the occurrence of the symptomatic disease, while its connection with neuroinvasion and intrathecal response in unclear.

Consistent with the previous studies, we have not observed an evident difference in the clinical presentation between TBE patients with *CCR5Δ32/wt* and *wt/wt* genotype neither in the group presented here nor in a larger population of genotyped patients from our center (unpublished data). There was also no difference in csf inflammatory parameters between *CCR5Δ32/wt* and *wt/wt* TBE patients. That suggests that either (1) CCR5 does not play a role in driving lymphocyte migration into cns in TBE, (2) CCR5 drives lymphocyte migration in TBE but its impaired expression may be compensated by alternative pathways, or (3) CCR5 drives lymphocyte migration in TBE, but its expression in *CCR5Δ32/wt* is induced to a level sufficient to fulfill that role. We attempted to clarify this by comparing a phenotypic CCR5 expression in TBE patients with both genotypes. As expected, the peripheral Th lymphocyte CCR5 expression in *CCR5Δ32/wt* heterozygotes was lower than in *wt/wt* homozygotes both in healthy controls and in TBE patients on admission to hospital, but in TBE, the difference became insignificant in the early convalescent period. This may be an artifact due to a small number of *CCR5Δ32/wt* samples, but if confirmed in a larger group of patients, it could also suggest a compensatory CCR5 up-regulation in response to TBEV challenge. More importantly, the CCR5 expression in csf obtained from three *CCR5Δ32/wt* TBE patients was several-fold increased in comparison with blood and reached the lower end of a value range found in *wt/wt* homozygotes. This suggest that the expression of CCR5 and/or its ligands was up-regulated to a level sufficient for a Th lymphocyte migration to cns and effective virus control, consistent with a normal pleocytosis and an unremarkable clinical presentation in *CCR5Δ32/wt* patients. CCL5 level in csf was not increased in *CCR5Δ32/wt* versus *wt/wt* genotype, so it seems not directly involved in a compensation for an impaired CCR5 expression. As *CCR5Δ32/wt* patients seem to have a normal intrathecal response to TBE, the increased susceptibility to TBEV associated with this genotype should depend on the impairment of the early, peripheral response, during the first febrile phase or even locally at the tick-bite site, which agrees with the conclusions from epidemiologic observations by Lim et al., who have suggested a similar mechanism for an increased susceptibility to WNV in *CCR5Δ32/CCR5Δ32* homozygotes [40].

*CCR5Δ32* variant is present in a small minority of TBE patients, including 2.3 % of *CCR5Δ32* homozygotes in the Lithuanian TBE group [41] and only 7 *CCR5Δ32/wt* heterozygotes in our study, so additional predisposing factors must be involved in the remaining cases. As evident in our results as well, the expression of CCR5 in peripheral blood T lymphocytes in *CCR5 wt/wt* homozygotes is highly variable. Several common genetic polymorphisms associated with that variability have been identified in the promoter region of *CCR5* and in the genes for CCR5 ligands, *CCL5* and *CCL3L1*, the latter probably influencing CCR5 expression indirectly, through their association with the CCL3 and CCL5 synthesis [44, 58, 59]. We have hypothesized that the variability of CCR5 expression associated with factors other than *CCR5Δ32* allele should have a similar clinical effect and contribute to a variable response to TBEV in the CCR5 *wt/wt* population. To verify that we have studied CCR5 in the peripheral blood Th cells in patients 6–8 weeks after the hospital admission for TBE, after the normalization of the clinical signs and symptoms and the inflammatory parameters in the periphery, we consider it a proxy of the constitutive baseline level. In fact, as we detected no hint of any dynamics of the peripheral CCR5 expression in CCR5 *wt/wt* homozygotes in the course of TBE, the values measured in the neurologic phase could be considered close to the baseline too. However, the median expression at any of the time points studied did not differ from the results in healthy controls and in other patient groups, which is especially striking in the face of a high individual variability. It could be speculated that TBE patients had originally lower CCR5 peripheral expression than controls, masked by a prolonged up-regulation lasting from the onset of meningitis/encephalitis to the late convalescent period. This assumption could be definitely dismissed only by a systematic study of patients with a history of TBE at still later time points, months to years after infection, to check if there is any sign of the return to a hypothetical lower baseline level. Alternatively, a study of CCR5 expression patients in a first phase of TBE before the onset of meningitis and the ones with a mild flu-like TBEV infection would be informative as for its role in the early response but is difficult to conceive as these patients are rarely diagnosed and hospitalized. Our observations with that respect are fragmentary: in three patients in whom peripheral blood lymphocyte CCR5 was measured in the first phase of the infection and who later developed meningitis, it was within the range of values found in other groups, as it was in three patients with a mild flu-like infection without cns involvement, not giving evidence for any inter-group variability or dynamical change of CCR5 expression before the cns invasion. At present, there is no data to support the association between the low constitutive CCR5 expression in the peripheral blood-activated Th cells and the susceptibility to TBEV in persons with a *CCR5 wt/wt* genotype.

## Conclusions

Our results support the pathogenetic role of CCR5 and its ligand CCL5 in the neurologic phase of TBEV infection. The expression of CCR5 in activated Th lymphocytes was not particularly high in TBE patients comparing with other viral and borrelial meningitis but was relatively long-lasting and correlated with other parameters of the intrathecal inflammation. Our study did not address the role of other chemokine receptors, which may be co-expressed and cooperate with CCR5. The possible co-expression of CCR5 and CXCR3 in the Th cell population and their relative importance for the lymphocyte migration warrants a further study, which could further clarify the role of CCR5 in this setting as well.

We did not detect evidently impaired CCR5 expression or any features of altered intrathecal inflammatory response in TBE patients heterozygous for *CCR5Δ32* allele, and we infer that the CCR5 expression is induced to a level adequate for the effective lymphocyte migration to cns in them. The observed increased incidence of TBE in *CCR5Δ32* bearers may depend on the impaired early peripheral response to TBEV, possibly even at the tick-bite site before the further virus spread, which requires further study. However, we found no evidence that, besides a relatively rare *CCR5Δ32* variant, a low baseline expression of CCR5 in the peripheral blood lymphocytes may be a marker of an increased susceptibility to flavivirus.
